# Developing the dielectric mechanisms of polyetherimide/multiwalled carbon nanotube/(Ba_0.8_Sr_0.2_)(Ti_0.9_Zr_0.1_)O_3 _composites

**DOI:** 10.1186/1556-276X-7-132

**Published:** 2012-02-16

**Authors:** Chean-Cheng Su, Chia-Ching Wu, Cheng-Fu Yang

**Affiliations:** 1 Department of Chemical and Materials Engineering, National University of Kaohsiung, Kaohsiung, 81148, Republic of China; 2 Department of Electronic Engineering, Kao Yuan University, Kaohsiung, 82151, Republic of China

**Keywords:** composites, mixing rule, dielectric properties, electrical conduction mechanism

## Abstract

Various amounts of multiwalled carbon nanotubes [MWNTs] were embedded into polyetherimide [PEI] to form PEI/MWNT composites, and their dielectric properties were measured at 1 MHz. The Lichtenecker mixing rule was used to find a reasonable dielectric constant for the MWNTs used in this study. The dielectric constants of the developed composites were significantly increased, and the loss tangents were significantly decreased as 2.0 wt.% (Ba_0.8_Sr_0.2_)(Ti_0.9_Zr_0.1_)O_3 _ceramic powder [BSTZ] was added to the PEI/MWNTs to form PEI/MWNT/BSTZ composites. The Lichtenecker and Yamada mixing rules were used to predict the dielectric constants of the PEI/MWNT and PEI/MWNT/BSTZ composites. Equivalent electrical conduction models of both composites were established using the two mixing rules. In addition, the theoretical bases of the two mixing rules were used to explain the measured results for the PEI/MWNT and PEI/BSTZ/MWNT composites.

## Introduction

Discovered accidentally by Sumio Iijima in 1991 [1], carbon nanotubes [CNTs] were a new form of carbon with unique physical, electrical, and mechanical properties. The CNTs can behave either as a semiconductor or as a metal and may have a number of practical applications. CNTs have also been embedded into polymers to fabricate composites with good electrical properties, including dielectric constants with higher values and good thermal stability [2]. In the present work, we investigate polymer/matrix composites with high dielectric constants using multiwalled carbon nanotubes [MWNTs] as fillers.

Polymer/ceramic composites with high dielectric constants have attracted much attention due to their simple, low-temperature processing and their flexibility. High-tech electronic devices require new materials with high dielectric constants, suitable dielectric properties, mechanical strength, and easy fabrication processes. Recently, polymer/ceramic composites have been studied in various applications, including integrated capacitors, acoustic emission sensors, and microwave substrates [3,4]. When BaTiO_3 _was used as a dielectric material, although it had a relative high dielectric constant (above 1,000), the effective dielectric constants of composites with high BaTiO_3 _content still remained relatively low due to the lower dielectric constant of the epoxy matrix. Bai et al. [5] reported a high dielectric constant for a polymer matrix composite containing a large amount of ferroelectric ceramic particles, which made the composite lose its flexibility. On the other hand, using metal particles as a filler yielded polymer/metal composites with high dielectric constants as only a small weight percentage of conductive particles was added, but the thermal stability of the dielectric constants was not good [6]. In previous reports, when the MWNTs were added to the polyetherimide [PEI] matrix and polyvinylidene fluoride/BaTiO_3 _composites, it enhanced the dielectric, thermal, and tensile properties of composites [7,8].

The ratio of the passive elements to active components in mobile communication, computer, and consumer electronic devices is over 20, and nearly 70% of the circuit board area is occupied by discrete capacitors. Because of that, the cost and size of an electronic device will apparently increase. To solve these problems, embedded capacitor technology, which incorporates capacitors into one of the inner layers of a multilayer substrate, has been investigated. The important requirements for embedded capacitor materials are high dielectric constant, low capacitance tolerance, and low cost. In the present study, the dielectric properties of PEI/MWNT composites were developed first for the possible applications in embedded capacitors. The imide groups provide strength at high temperatures, while the flexible ether group linkages support a relatively easy processing. The properties of the MWNTs were similar to those of metals, and high dielectric constants were obtainable for the polymer/MWNTs with just a small weight percentage of MWNTs. The Lichtenecker mixing rule was used to find a reasonable dielectric constant for the MWNTs used in this study. (Ba_0.8_Sr_0.2_)(Ti_0.9_Zr_0.1_)O_3 _[BSTZ] has a higher dielectric constant, lower dielectric loss, and broader dielectric peak [9], so BSTZ was added to the PEI/MWNT (MWNTs = 2.0 wt.%) composites to increase the dielectric constants and decrease the loss tangents of the PEI/MWNT composites. Finally, the Lichtenecker and Yamada mixing rules were used to predict the dielectric constants of the PEI/MWNT and PEI/MWNT/BSTZ composites.

## Experimental details

A 125-ml round-bottom flask equipped with a condenser and a stirrer was charged with MWNTs, sulfuric acid (98%), and nitric acid (63%). The flask was sonicated for 30 min using an ultrasonic apparatus, and chemical oxidation was carried out at 60°C for 48 h. The diameter distribution of functionalized CNTs was 20 to 50 nm, and the length distribution was 2 to 15 μm. The MWNTs were functionalized with carboxylic acid groups (COOH) on their surfaces. BaCO_3_, SrCO_3_, TiO_2_, and ZrO_2 _were mixed to achieve the BSTZ ceramic. The powder was calcined at 1,100°C for 2 h; the calcined powder was uniaxially pressed into pellets, and then the pellets were sintered at 1,450°C for 2 h. Next, the ceramic was ground into a fine powder; the particle size distribution was 1 to 5 μm, and the average particle was 3 μm. Using an ultrasonic cleaner, the neat PEI was dissolved in dichloromethane [CH_2_Cl_2_] solvent, and the MWNTs were mixed with a solution of PEI and CH_2_Cl_2 _to form the PEI/MWNT composites. The PEI/MWNT/BSTZ composites were prepared using a special methylene chloride solvent mixing method, and commercial KD1 dispersant was added. The MWNTs and BSTZ ceramic powder in PEI matrix solutions were cast in a rotation mold at 60°C, and the residual solvent was vaporized in a vacuum at 60°C for 24 h. Fourier transform infrared [FTIR] spectra were used to identify the functional groups responsible for the chemical modification of the MWNTs. The morphologies of the PEI/MWNT/BSTZ composites were observed from scanning electronic micrographs [SEM]. The dielectric constants (*ε*_r_) and loss tangents (tan*δ*) of the PEI/MWNT and PEI/MWNT/BSTZ composites were measured at 1 MHz using an LCR meter HP 4294 (Agilent Technologies Inc., Santa Clara, CA, USA).

## Results and discussion

**Figure 1:**
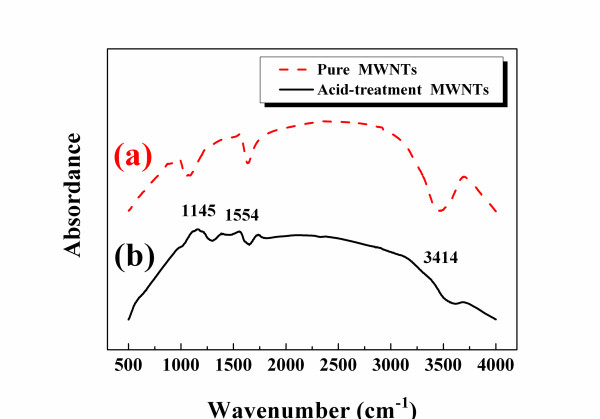
**FTIR analysis of MWNTs**. (a) Pure MWNTs and (b) acid-treated MWNTs.

Figure 1 shows the FTIR spectra in the range of 500 to 4,000 cm^-1 ^of the MWNTs and the acid-treated MWNTs. Both spectra in Figure 1a, b include main absorption peaks that are characteristic of the hydroxide group in the 3,200 to 3,700 cm^-l ^range, absorption by the carboxyl group at 1554 cm^-1^, and C-O stretching vibrations at 1,145 cm^-1 ^[10]. Figure 1a shows that the original MWNTs yielded a more intensive hydroxyl absorption peak at 3,570 cm^-l ^than the treated MWNTs. Figure 1b shows that the intensities of the main characteristic absorption peaks and of the carboxyl group and C-O stretching vibrations for the chemically modified MWNTs were more obvious. However, these results suggest that carboxylic acids rather than hydroxyl groups were covalently attached to the *π*-conjugated skeleton of the MWNTs. Compared with the unmodified MWNTs, the surfaces of the chemically modified MWNTs have more attached polar functional groups, such as -OH, -C = O, and -C-O, to improve their interface interaction with the PEI matrix.

**Figure 2:**
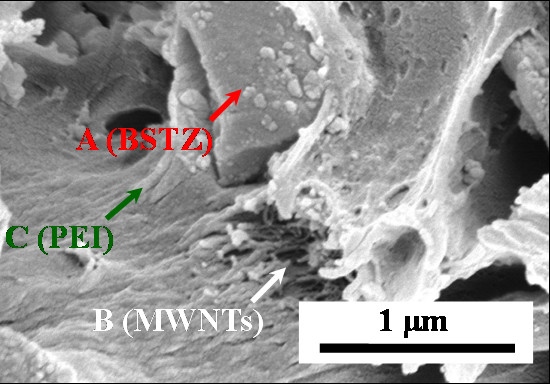
**Cross-section SEM images of the PEI/MWNT/BSTZ composites**.

The cross-section SEM morphology of the PEI/MWNT/BSTZ composites is shown in Figure 2, where arrows A, B, and C indicate BSTZ, MWNTs, and PEI, respectively. The 2 wt.% MWNTs and 60 wt.% BSTZ were effectively dispersed in the PEI matrix using ultrasonic waves. The good dispersion of the MWNTs was due to strong interfacial interactions and chemical compatibility between the PEI matrix and the functionalized MWNTs, caused by the strong interactions between the carboxyl and hydroxyl groups of the MWNTs, and the N and O of the PEI molecules [11].

**Figure 3:**
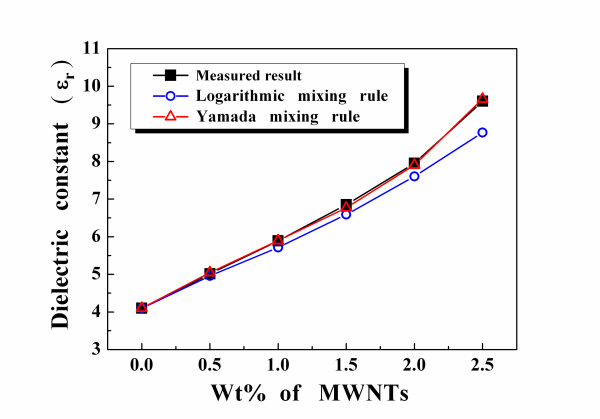
**Measured and predicted dielectric constants of the PEI/MWNT composites**.

Figure 3 plots the measured and predicted dielectric properties of the PEI/MWNT composites. As what Figure 3 shows, when the MWNT content increased from 0 to only 2.5 wt.%, the dielectric constant of the PEI/MWNT composites increased from 3.9 to 9.7. Predicting the dielectric constants of polymer/filler composites is important to develop them for new applications, and we used two mixing rule equations to predict the dielectric constants of the PEI/MWNT composites. The Lichtenecker mixing rule is extensively applied to composites with *m *components, with half in parallel and half in series, and this is the general form of the equation [12]:

(1) $$\text{log}\varepsilon =\overset{m}{\underset{i=1}{\sum }}v_{i}\text{log}\varepsilon_{i},$$

where *v_i _*and *ε_i _*represent the volume fraction and the dielectric constant of each material, respectively, and $\sum v_{i}=1$. Yamada et al. [13] studied on the assumption that a binary system is composed of ellipsoidal particles dispersed in a continuous medium; the dielectric constants of the composites are given using the following equation:

(2) $$\varepsilon =\varepsilon_{1}\left[1+\frac{v_{2}\left(\varepsilon_{2}-\varepsilon_{1}\right)}{\varepsilon_{1}+n\left(\varepsilon_{2}-\varepsilon_{1}\right)\left(1-v_{2}\right)}\right],$$

where *n *= 1/*η *is the morphology factor, which depends on the shape of the particles and their orientation relative to the composite surfaces. The morphology factor is any number between 0 and 1, with 0 representing all connections in parallel and 1 representing all connections in series.

First, the Lichtenecker mixing rule was used to find a reasonable dielectric constant of the MWNTs from the measured dielectric constants of the PEI/MWNT composites. The reasonable dielectric constant of the MWNTs was approximately 10^15^, and the conductivity was conjectured to be similar to that of a metal. The measured dielectric constants of the PEI/MWNT composites were compared with the predicted results from the two mixing rules, with *ε*_PEI _= 4.1 and *ε*_MWNTs _= 10^15^. According to the measurements, the *η *value of the Yamada equation was not constant. Therefore, an attempt was made to evaluate this parameter from the measured dielectric constants of the PEI/MWNT composites. As we know, the morphology factor of the PEI/MWNT composites changed from 0.473 to 0.271 as the MWNT content increased. Figure 3 also shows that the measured dielectric constants of the PEI/MWNT composites with higher MWNT content are different from the Lichtenecker-predicted results but agree closely with the Yamada-predicted results.

**Figure 4:**
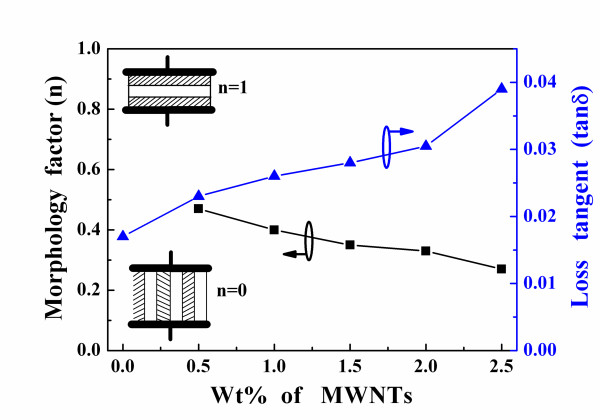
**Loss tangents and morphology factor of the PEI/MWNT composites**.

These outcomes indicate that as the MWNT content increased, the degree of parallel connection in the microstructures of the PEI/MWNTs also increased, whereas the degree of series connection decreased. In Figure 3, the errors between the measured and the Yamada-predicted dielectric constants are less than those between the measured and the Lichtenecker-predicted results, especially when the MWNT content is more than 1.5 wt.%. The Lichtenecker mixing rule assumes that fillers are uniformly distributed in a matrix. However, a homogeneous distribution in the polymer matrix is very difficult to achieve because the high dielectric constants of MWNTs are not very well dispersed in the low dielectric constant PEI matrix. The loss tangents of the PEI/MWNT composites were less than 4%, as shown in Figure 4. In this study, PEI/2 wt.% MWNT composites were chosen for future applications in integrated passive capacitance devices or microwave substrates because their loss tangents were less than 3%.

**Figure 5:**
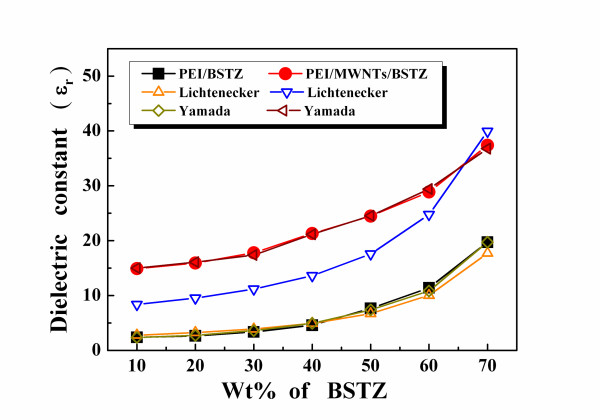
**Measured and predicted dielectric constants of PEI/BSTZ and PEI/MWNT/BSTZ composites as function of BSTZ content**.

Figure 5 shows the dielectric constants of the latter composites that are increased from 14.2 to 35.8 as the BSTZ content increased from 10 to 70 wt.%. Compared with the PEI/BSTZ composites, the dielectric constants of the PEI/MWNT/BSTZ composites were significantly better when 2.0 wt.% MWNTs was added. Figure 5 also shows that differences exist between the measured and predicted dielectric constants of the PEI/2wt.% MWNT/BSTZ composites when *ε*_PEI _= 3.9, *ε*_BSTZ _= 3,000, and *ε*_CNTs _= 10^15 ^are used. The Yamada equation uses PEI/2 wt.% MWNTs and BSTZ as two phases to predict the dielectric constants of the PEI/2wt.% MWNT/BSTZ composites. The errors between the measured and the Lichtenecker-predicted dielectric constants are larger than those between the measured and the Yamada-predicted results.

**Figure 6:**
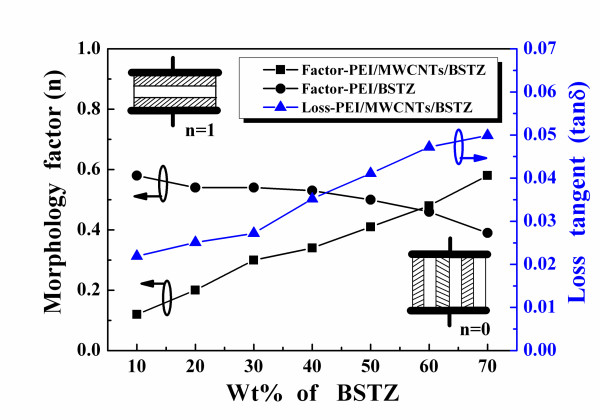
**Loss tangents and morphology factors of the PEI/MWNT/BSTZ composites**.

Figure 6 reveals that the morphology factors of the PEI/BSTZ composites decreased from 0.40 to 0.59 as BSTZ content increased from 10 to 70 wt.%, with an average value of 0.53. This demonstrates that the BSTZ ceramic powder was more uniformly distributed in the PEI matrix to form the composites. The morphology factor of the PEI/2 wt.% MWNT/BSTZ composites increased from 0.06 to 0.58 as the BSTZ content increased from 10 to 70 wt.%, indicating that the microstructures of the PEI/MWNT/BSTZ composites with lower BSTZ powder content were closely in parallel, which decreased as the BSTZ content increased. As the BSTZ content was less than 70 wt.%, the fact that the Lichtenecker-predicted dielectric constants are smaller than the measured results (Figure 5) proves the theory. The conductivity materials of MWNTs are similar to metal and would have been equivalent to an electrode in the PEI/2 wt.% MWNT/BSTZ composites during the measurements. Therefore, more parallel connection microstructures existed in the PEI/MWNT/BSTZ composites with lower BSTZ content. This result was due to the PEI/MWNT/BSTZ composites with lower BSTZ content having larger dielectric constants than the predicted values obtained using the Lichtenecker mixing rule. A higher BSTZ powder content reduced the number of parallel connections in the PEI/MWNT/BSTZ composites. Therefore, the dielectric constants of the PEI/2 wt.% MWNT/BSTZ composites are close to the predicted values from the Lichtenecker mixing rule. In Figure 6, the loss tangents of the PEI/MWNT/BSTZ composites are less than 5%. These results suggest that PEI/MWNT/BSTZ composites are good candidates to develop for future electric devices, and the optimum BSTZ powder contents are 60 and 70 wt.% in the PEI/MWNT/BSTZ composites.

**Figure 7:**
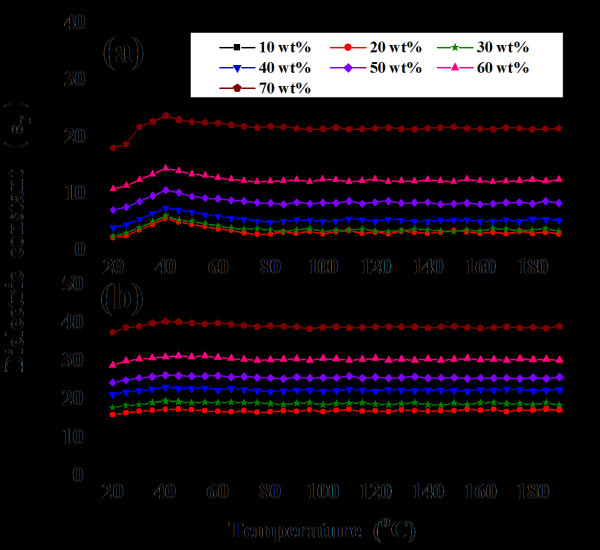
**Dielectric constants of (a) PEI/BSTZ and (b) PEI/2 wt**.% MWNT/BSTZ composites as function of temperature.

The dielectric constant-temperature curves of the PEI/BSTZ and PEI/2 wt.% MWNT/BSTZ composites are shown in Figure 7. The dielectric peak of the PEI/BSTZ composites around 40°C (the *T*_c _of BSTZ ceramic) is not discernible in the PEI/2 wt.% MWNT/BSTZ composites. In addition, compared to polymer composites filled with metal particles [14], the PEI/2 wt.% MWNT/BSTZ composites had dielectric constants with good thermal stability. The mentioned result indicates that the dielectric constants of the PEI/2 wt.% MWNT/BSTZ composites with different BSTZ contents (10 to 70 wt.%) remained almost constant up to a temperature of 190°C. In comparison with other reports, the PEI/MWNT/BSTZ composites get the lower loss tangents and better thermal stability of dielectric constants in this study.

## Conclusions

In this investigation, a conductivity material in the form of MWNTs and a ferroelectric material in the form of BSTZ ceramic powder were added to a PEI matrix to form PEI/MWNT and PEI/MWNT/BSTZ composites. The dielectric constants of the PEI/MWNT composites increased from 3.9 to 9.7 as the MWNT content increased from 0 to 2.5 wt.%. The dielectric constants of the PEI/2 wt.% MWNT/BSTZ composites increased from 14.2 to 35.8 as the BSTZ content increased from 10 to 70 wt.%. The loss tangents of all the PEI/2 wt.% MWNT/BSTZ composites measured at 1 MHz were less than 0.05. Using the Lichtenecker and Yamada mixing rules, equivalent electrical conduction models of the PEI/MWNT and PEI/2 wt.% MWNT/BSTZ composites were established. The results indicate that these PEI/MWNT and PEI/2 wt.% MWNT/BSTZ composites are attractive materials for applications in electrical devices.

## Competing interests

The authors declare that they have no competing interests.

## Authors' contributions

C-CS participated in the fabrication of composites, MWNT functionalization, and FTIR analyses. C-CW participated in the fabrication of composites, physical analyses, and electrical measurements. C-FY participated in electrical measurements and prediction of dielectric constants using the Lichtenecker and Yamada equations. All authors read and approved the final manuscript.
